# Supplementary material for: Modulation of human dorsal root ganglion neuron excitability by Nav1.7 inhibition
Source: eLife. 2026 Oct 23;15:RP111490. doi: 10.7554/eLife.111490 (PMC13694097; doi:10.7554/eLife.111490)
Supplement: Peer review history

# Editor report: eLife Assessment

**DOI:** 10.7554/eLife.111490.3.sa0

**Authors:** Murali Prakriya

**Affiliations:** Northwestern University United States

**Keywords:** Convincing, Important

This **important** study investigates the role of Nav1.7 voltage-gated sodium channels in regulating excitability of human dorsal root ganglion (hDRG) neurons. The authors characterize a previously identified Nav1.7 channel inhibitor using recombinant channels and human neurons. The study **convincingly** shows that inhibition of Nav1.7 channels with AM-2099 causes a modest decrease in neuronal excitability and prolongs the refractory period. This work offers new insights into the mechanism of clinically relevant pharmacological targets for pain relief.

# Referee report: Reviewer #1 (Public review)

**DOI:** 10.7554/eLife.111490.3.sa1

Summary:

Fujita and colleagues investigated two selective peripheral nerve voltage-gated sodium channel inhibitors targeting either Nav1.7 or Nav1.8 on excitability of human dorsal root ganglion neurons. The authors discovered that Nav1.8 inhibition is more effective at suppressing repetitive firing of DRG neurons and this may explain the greater clinical efficacy observed for suzetrigine.

Strengths:

The study is interesting and the findings are conceptually satisfying in that they may explain one aspect of Nav1.7 vs Nav1.8 targeting success.

Weaknesses:

(1) The use of postmortem human DRG neurons provides translational relevance, but the use of these cells is also a liability given their high degree of variability. Of note are the 10 to 20-fold differences in baseline properties among cells, which dwarfs the effects of the test compounds. The experiments may suffer from under sampling.

Comments on revised version.

The revised manuscript addresses my prior concern with reasonable effort given the limitations of human postmortem DRGs.

# Referee report: Reviewer #3 (Public review)

**DOI:** 10.7554/eLife.111490.3.sa2

Summary:

In this manuscript, Fujita/Jo/Stewart/Osorno et al., investigate the contribution of Nav1.7 in regulating the excitability and firing properties of human dorsal root ganglion (hDRG) neurons in vitro. The authors characterize the effects of a previously reported Nav1.7-selective blocker AM-2099 in recombinant human Nav1.7 channels and in cultured hDRG neurons from postmortem organ donors. The authors observed modest changes in many of the properties expected by inhibiting Nav channels, including decreased action potential upstroke rate and amplitude, while increasing the voltage and current thresholds for spike generation. However, AM-2099 did not change the maximum number of APs in response to suprathreshold stimulation, leading the authors to conclude that Nav1.7 inhibition alone has limited efficacy in reducing the firing properties of hDRG neurons at the soma, and discuss that the effects of Nav inhibition may be different at distal axons.

Strengths:

Experiments are well-designed and executed, and the results presented are convincing. The focus on voltage-gated sodium channels in native human DRG neurons is highly relevant to recent efforts to develop safer analgesic options for chronic pain in people.

Comments on revised version.

The authors have done an excellent job addressing my prior critiques.

# Author comment: Author response

**DOI:** 10.7554/eLife.111490.3.sa3

**Authors:** Akie Fujita, Sooyeon Jo, Robert G Stewart, Tomás Osorno, Alyssa Ferraiuolo, Kevin Carlin, Bruce P Bean

**Affiliations:** Harvard Medical School Boston United States; Harvard Medical School Boston United States; Harvard Medical School Boston United States; Harvard Medical School Boston United States; AnaBios Corporation San Diego United States; AnaBios Corporation San Diego United States; Harvard Medical School Boston United States

The following is the authors’ response to the previous reviews.

**Public Reviews:**

**Reviewer #1 (Public review):**

Summary:

Fujita and colleagues investigated two selective peripheral nerve voltage-gated sodium channel inhibitors targeting either Nav1.7 or Nav1.8 on the excitability of human dorsal root ganglion neurons. The authors discovered that Nav1.8 inhibition is more effective at suppressing repetitive firing of DRG neurons, and this may explain the greater clinical efficacy observed for suzetrigine.

Strengths:

The study is interesting, and the findings are conceptually satisfying in that they may explain one aspect of Nav1.7 vs Nav1.8 targeting success.

Weaknesses:

(1) The use of postmortem human DRG neurons provides translational relevance, but the use of these cells is also a liability, given their high degree of variability. Of note are the 10 to 20-fold differences in baseline properties among cells, which dwarf the effects of the test compounds. The experiments may suffer from undersampling.

We have added data from an additional 3 donors for the key results on increase in threshold and reduction of action potential upstroke, more than doubling the number of neurons for this data. We have also added a Supplementary Figure (Figure S2) that breaks out the effects on these parameters for each donor. This illustrates that there is a high degree of neuron-to-neuron variability in the effect of inhibiting Nav1.7 channels even within a single donor, even though we confined data to neurons that were verified to be capsaicin-sensitive. We also now note that there is a similar high degree of cell-to-cell variability in relative functional expression of Nav1.7 and Nav1.8 channels in capsaicin-sensitive mouse DRG neurons.

(2) A potential confounder when using post-mortem human DRG neurons is heterogeneity of cell types. The methods clearly state that the cells selected for recording were of 'generally' small size, but specific criteria for what constitutes 'small' or other unstated selection criteria were not provided. A table of individual cell capacitance and input resistance values, along with information about individual donors (age, sex, ethnicity), is important to include. Additionally, some discussion of how DRG neuron heterogeneity impacts the findings. This relates to concern #1 about sample size determination and how cell heterogeneity factored into this calculation.

We have added a figure (Figure S1) showing histograms and box plots of individual cell capacitance, input resistance, resting potentials, and maximum upstroke. We have also added a table with the information about donors (Table S1). As noted, we have also added a figure (Figure S2) that breaks out the effects on these parameters for each donor, illustrating that there is a high degree of neuron-to-neuron variability in the effects even within a single donor. We have added several sentences to the Discussion concerning the neuron-to-neuron variability in the effects of Nav1.7 inhibition, including the possibility that this may reflect heterogeneity of cell function.

**Reviewer #2 (Public review):**

Summary:

The authors examine the functional role of Nav1.7 voltage-gated sodium channels in human sensory neuron electrogenesis using a Nav1.7 selective inhibitor and human dorsal root ganglion neurons obtained from organ donors. Patch-clamp electrophysiology is used at physiological temperature to measure the impact of Nav1.7 inhibition on sensory neurons' action potential firing. This is an important topic as Nav1.7 and Nav1.8 have been identified as therapeutic targets for the treatment of pain, but there has been mixed success with isoform-specific inhibitors in clinical trials. The data suggest that Nav1.7 and Nav1.8 have overlapping yet complementary functions in nociceptor neurons and that targeting both may be most effective for reducing nociception.

Strengths:

The data are of high quality. Action potential properties are measured at 37 degrees Celsius. Threshold is measured using brief pulses. The Nav1.7 inhibitor has been reported to be highly selective for Nav1.7 over Nav1.8 and moderately selective for Nav1.7 over Nav1.1 and Nav1.6. Data are collected using identical conditions and protocols to a previous study on the role of Nav1.8 in similar neurons.

Weaknesses:

The study relies on a single Nav1.7 inhibitor that has not been extensively characterized. One prior study indicates that the IC50 is around 140 nM, thus the 600 nM concentration used in this study could be predicted to reduce Nav1.7 currents by 80%. However, there is no voltage-clamp data in the current study to confirm this, and therefore, it is unclear if the batch of AM-2099 is as potent as reported in the paper that initially described its selectivity. The impact of Nav1.7 inhibition is compared to data from a previous study by this lab, and this is a minor concern. It would have been interesting to see if the combined inhibition of Nav1.7 and Nav1.8 completely blocked action potential generation in the human DRG neurons.

We have done experiments to directly characterize the potency of the AM-2099 sample we used on both cloned human Nav1.7 channels and on native currents in the DRG neurons. Using a stable cell line expressing human Nav1.7 channels, we determined dose-response curves at both 22°C and 37°C, using an automated patch clamp instrument. These results are shown in a new Figure 1. Interestingly, we found that the IC_50_ is substantially higher at 37°C than at room temperature. We also did experiments quantifying the effect of 100 nM and 600 nM AM-2099 on native sodium currents in the human DRG neurons, which align well with the results on the cloned Nav1.7 channels in suggesting that at 37°C, 600 nM AM-2099 inhibits Nav1.7 channels by about 85%.

We have also added a new figure (Figure S3) showing the effects of a different Nav1.7 inhibitor, PF04856264. The effects of this inhibitor were qualitatively identical but quantitatively smaller than those of AM-2099. When we realized this, we did voltage clamp experiments on cloned Nav1.7 channels and discovered that the potency of PF-04856264 at 37°C was weaker than expected from the published IC_50_, which was determined at room temperature.

We are currently doing experiments testing combined inhibition of Nav1.7 and Nav1.8 channels whenever we can obtain human neurons. Because it is of interest to examine effects of partial as well as full inhibition of each channel type, there are multiple permutations of inhibitors combined and alone that are of interest to characterize, and these studies are still in progress. We think the results in the present manuscript stand on their own and together with previous data on effects of Nav1.8 inhibitors alone provide a foundation for on-going and future studies on combinations of inhibitors by ourselves and others.

**Reviewer #3 (Public review):**

Summary:

In this manuscript, Fujita/Jo/Stewart/Osorno et al. investigate the contribution of Nav1.7 in regulating the excitability and firing properties of human dorsal root ganglion (hDRG) neurons in vitro. The authors characterize the effects of a previously reported Nav1.7-selective blocker AM-2099 in cultured hDRG neurons from postmortem organ donors. The authors observed modest changes in many of the properties expected by inhibiting Nav channels, including decreased action potential upstroke rate and amplitude, while increasing the voltage and current thresholds for spike generation. However, AM-2099 did not change the maximum number of APs in response to suprathreshold stimulation, leading the authors to conclude that Nav1.7 inhibition alone has limited efficacy in reducing the firing properties of hDRG neurons and that Nav1.7 blockers may have limited efficacy as analgesics. This is surprising, given that patients with loss-of-function mutations in Nav1.7 suffer from congenital insensitivity to pain. While it may indeed be true that pharmacological inhibition of Nav1.7 is unlikely to produce analgesia, the present study was limited to a single concentration of AM-2099. The manuscript would be significantly strengthened by a more careful and thorough pharmacological characterization of this compound, which has not been widely used or validated in native human DRG neurons.

Strengths:

Experiments are well-designed and executed, and the results presented are convincing. The focus on voltage-gated sodium channels in native human DRG neurons is highly relevant to recent efforts to develop safer analgesic options for chronic pain in people.

Weaknesses:

Only a single concentration of AM-2099 was used for all experiments. This compound was reported to be selective for cloned human Nav1.7 channels in heterologous systems, but has not been validated in other studies after the original publication in 2016. Since the original study reported a substantial statedependent block of recombinant Nav1.7 channels, more detailed pharmacological characterization of AM-2099 is needed in human DRG neurons to fully support these claims. This study would be significantly strengthened by the inclusion of dose-response curves to assess how much of the sodium current is inhibited at this concentration, confirming selectivity in hDRG, and whether maximal inhibition of Nav1.7 still has limited efficacy in reducing the firing of native human sensory neurons.

We have added results from experiments to directly quantify the potency of AM-2099 on both cloned human Nav1.7 channels (new Figure 1) and on native currents in the DRG neurons (new Figure 2). These show that 600 nM AM-2099 produces about 85% inhibition of Nav1.7 channels at 37°C. We have added a paragraph to the Discussion explaining that we chose this concentration of AM-2099 to produce reasonably complete inhibition of Nav1.7 channels while minimizing potential inhibition of a component of non-Nav1.7 TTX-sensitive current.

With regard to the broader point about reconciling the variable and sometimes relatively modest effects of Nav1.7 inhibition with the complete loss of pain sensation in humans with loss-of-function mutations, we have modified the Introduction and Discussion to eliminate any implication that the results in the manuscript suggest that pharmacological inhibition of Nav1.7 is unlikely to produce analgesia. Our experiments are only on action potential firing in the cell body, and it is perfectly possible that inhibiting Nav1.7 channels in the axon could disrupt generation or propagation of action potentials, either in the main axon or in the fine axon terminals in the spinal cord. We have modified the Discussion to explicitly point this out, which would reconcile the loss of pain sensation in humans with loss-offunction mutations with the incomplete effects of Nav1.7 inhibitors on excitability of cell bodies.

**Recommendations for the authors:**

**Reviewing Editor comments:**

In addition to the points noted in the eLife assessment summary above, the study has several important strengths, including use of human primary neurons and recordings performed under physiologically relevant conditions (at 37 {degree sign}C using brief current injections). However, reviewers also identified several key weaknesses that must be addressed to support the central conclusions. In particular, multiple reviewers raised concerns regarding the lack of voltage-clamp data evaluating the efficacy and specificity of AM-2099 inhibition of Nav1.7 currents. A single dose of 600 nM was used based on the report of Marx (2016) in recombinant systems (Marx, 2016). Since no other studies other than the single Amgen report exist on this compound, it is important to validate its effects directly in the human DRGs used here. Additional concerns include the lack of dose-response analysis, as well as the large variability in baseline properties, which complicates the interpretation of the results. To assist the revision of the study, we outline below the key issues that should be addressed.

Recommendations for authors:

(1) Add voltage-clamp experiments to directly measure Nav1.7 current inhibition by AM-2099 in hDRG neurons. Given the limited previous characterization of this compound, it is important to confirm that the concentration used here (600 nM) effectively blocks Nav1.7 currents in the native system used here.

(2) Related to point 1 above, perform a dose-response of AM-2099 on hDRGs on Nav1.7 currents in human DRGs. Since this study, at least in part, is framed as a comparative analysis of Nav1.7 vs Nav1.8 channel subtypes in DRGs, it seems important to establish pharmacological equivalence to ensure that the comparisons are made at functionally comparable levels of channel block.

We have added results from experiments to directly characterize the potency of AM-2099 on both cloned human Nav1.7 channels and on native currents in the DRG neurons. Using a stable cell line expressing human Nav1.7 channels, we determined dose-response curves at both 22°C and 37°C, using an automated patch clamp instrument. These results are shown in a new Figure 1. Interestingly, we found that the IC^50^ is substantially higher at 37°C than at room temperature. We also did experiments quantifying the effect of 100 nM and 600 nM AM-2099 on native sodium currents in the human DRG neurons, which align well with the results on the cloned Nav1.7 channels in suggesting that at 37°C, 600 nM AM-2099 inhibits Nav1.7 channels by about 85%.

(3) Reviewer 1 notes that there seem to be 10-20-fold differences in baseline firing properties, which would exceed the effects of the test compound. This raises concerns about undersampling. Additional analysis or experiments would strengthen the conclusions.

We have added data from an additional 3 donors for the key results on increase in threshold and reduction of action potential upstroke, more than doubling the number of neurons for this data. We have also added a Supplementary Figure that breaks out the effects on these parameters for each donor. This illustrates that there is a high degree of cell-to-cell variability in the effects even within a single donor, even though we confined data to neurons that were verified to be capsaicin-sensitive. Reviewer 1 made the excellent suggestion that because of the neuron-to-neuron variability in baseline properties, the effects of compounds could be better illustrated by displaying changes from baseline. Following this suggestion, we have added Tukey-style box plots displaying the data in this way. Together with the donor-to-donor breakout of data in the new Figure S2, these plots make it clear that the neuron-to-neuron variability reveals genuine differences in the channel make-up of each neuron and not experimental error.

(4) Reviewer 2 notes an interesting experiment: does a combined block of Nav1.7 with the AM compound and Nav1.8 block action potential generation? If Nav1.7 controls threshold and Nav1.8 controls firing, then the combined inhibition should be highly effective in blocking nociceptive output, which could have therapeutic relevance.

We are currently doing experiments testing combined inhibition of Nav1.7 and Nav1.8 channels whenever we can obtain human neurons. Because it is of interest to examine effects of partial as well as full inhibition of each channel type, there are multiple permutations of inhibitors combined and alone that are of interest to characterize, and these studies are still in progress. We think the results in the present manuscript stand on their own and together with previous data on effects of Nav1.8 inhibitors alone provide a foundation for ongoing and future studies on combinations of inhibitors by ourselves and others.

**Reviewer #1 (Recommendations for the authors):**

Concerns in addition to those in the Public Review:

Major:

(1) As per point 1 of the weaknesses in the Public Review, I'm concerned that the experiments suffer from undersampling. This requires a discussion of how the sample size was determined.

We have added experiments from an additional 3 donors to the key results in Figures 3-5, more than doubling the number of neurons for these measurements.

(3) The effect of compounds could be better displayed as a change from baseline in Figure 1C-E. Also, are the AP traces and phase plots shown in Figures 1AB and 2AB averages or representative?

Thanks for this excellent suggestion. We have added box-plots that show changes from baseline for the various parameters. We have also clarified that the action potential traces and phase plots are from application of AM-2099 in a single representative neuron.

Minor:

(1) Provide source of VX-548 and report the purity of both compounds.

We have provided the information for VX-548 and added the information on the purity of both compounds

(2) Clinical failures of Nav1.7 blockers may not be solely due to pharmacodynamic limitations as implied by this study. Pharmacokinetic differences and toxicity (e.g., effects on the autonomic nervous system) may also have contributed.

Thanks for raising this important point. We have added this point to the Introduction.

**Reviewer #2 (Recommendations for the authors):**

It is an interesting study, and the conclusions are reasonable. However, it would have been good to see validation of the potency of AM-2099 on native DRG sodium currents and/or recombinant human Nav1.7 channels expressed in a heterologous expression system.

We have added results from experiments to directly quantify the potency of AM-2099 on both cloned human Nav1.7 channels (new Figure 1) and on native currents in the DRG neurons (new Figure 2).

Minor comments:

(1) Page 3, middle paragraph - there is a "(" missing before Renganathan.

Thanks, corrected.

(2) Page 4: Is anything known about AM-2099 in terms of state-dependence? It seems like Marx 2016 is the only previously published study using it, so additional information on the inhibitor would be helpful.

We have not characterized the state-dependence of AM-2099, but we characterized its potency in voltage clamp using holding voltages similar to the average resting potentials of the cells in current clamp conditions.

(3) Page 6 discusses that there might be differences between human and rodent DRG neurons in terms of Nav1.7 and Nav1.8. It would be nice if this were directly tested with these same Nav1.7 and Nav1.8 inhibitors.

We have recently done such a study on mouse DRG neurons which has just been published (J Physiol. 604:6104-6127, doi: 10.1113/JP290574).

(4) Figure 2A, right panel: I could not figure out the difference between the red and green traces. Perhaps this could be explained in the figure legend?

Thank you for pointing out that this was confusing. These two traces showed two different subthreshold responses, one of which was slightly regenerative without generating a full-blown spike. We have simplified the figure by now showing only a single subthreshold response.

**Reviewer #3 (Recommendations for the authors):**

(1) The conclusion that Nav1.7 inhibition has limited efficacy for inhibiting the firing of human DRG neurons is not fully supported by the data. This may be true, but it cannot be concluded without a more thorough pharmacological characterization of this compound. Dose-response curves and experimental confirmation of Nav1.7 selectivity (maybe just total Nav current, TTX-sensitive and TTX-resistant components) are needed.

We agree and have now added two new figures with this data.

(2) How was the 600 nM concentration chosen? Given that AM-2099 was reported to exhibit state dependent block, how much of the Na current is inhibited by this concentration at the initial voltage used in current clamp experiments (~-80 mV)?

We have added a paragraph to the Discussion recognizing the limitation that 600 nM AM-2099 produces ~85% rather than complete inhibition of Nav1.7 current and explaining that we chose this concentration of AM-2099 to produce reasonably complete inhibition of Nav1.7 channels while minimizing potential inhibition of a component of non-Nav1.7 TTX-sensitive current.

(3) It appears that the effects of AM-2099 on the refractory period are bimodally distributed, where neurons that recovered more slowly at baseline were preferentially affected by AM-2099 (Figure 4). Do these reflect different neuronal populations (e.g. smaller or larger diameter DRG) or different resting voltages in these experiments?

We agree that there seem to be two groups based on initial refractory period. Examining the parameters for the cells, there is no clear correlation between the effects of AM-2099 on the refractory period with resting potential or cell diameter. At this time, it is not obvious what determines the differences in refractory period. We speculate that neuron-to-neuron differences in the potassium conductances that generate the after hyperpolarization may be different in these cells but it will take further work to explore this.

(4) How much of the sodium current is mediated by Nav1.7 in hDRG neurons? How does inhibition of both Nav1.7 and Nav1.8 affect hDRG excitability?

The new Figure 2 shows data quantifying the AM-2099-sensitive current in the DRG neurons. With regard to combined Nav1.7 and Nav1.8 inhibition, we are currently doing experiments examining inhibition of excitability by combined Nav1.7 and Nav1.8 inhibition, which we agree is the logical next step in exploring how the two components of current control excitability. These are still in progress. Because designing and interpreting these experiments is facilitated by the current experiments with Nav1.7 inhibition alone, we believe that reporting the current results now will serve the scientific community better than waiting to obtain and interpret a body of data on dual inhibition in a sufficient number of donors, which we obtain only sporadically.

(5) Donor information and soma diameters should be included. Capsaicin sensitivity testing was mentioned in the methods, but I was unable to find any inclusion of these data in the results. These may be useful to potentially infer effects in different cell types.

We have added a figure (Figure S1) showing histograms and box plots of individual cell capacitance, input resistance, resting potentials, and maximum upstroke. We have also added a table with the information about donors (Table S1). We have now clarified that data were confined to cells verified to be capsaicin-sensitive and that ~95% of all cells tested were capsaicin-sensitive.

(6) Please check statistical tests and reporting. Several graphs do not appear to have paired responses (e.g. Figure 1E, Figure 4B). As a result, two-tailed Wilcoxon tests would not be appropriate. Also, check reported p-values (e.g. p=.0002), which are identical for multiple panels in the Results section.

We have clarified that the symbols of action potential width in control without a corresponding value after AM-2099 represent neurons in which the action potential in AM-2099 had a peak < 0 mV. These cells were not included in the data set of paired parameters used for the Wilcoxon test. We have also checked and verified all statistical tests.
